# Metagenomics of Virus Diversities in Solid-State Brewing Process of Traditional Chinese Vinegar

**DOI:** 10.3390/foods11203296

**Published:** 2022-10-21

**Authors:** Zhen Yu, Yan Ma, Yingfen Guan, Yuanyuan Zhu, Ke Wang, Yuqin Wang, Peng Liu, Juan Chen, Yongjian Yu

**Affiliations:** 1 School of Grain Science and Technology, Jiangsu University of Science and Technology, Zhenjiang 212100, China; 2 College of Food Science and Engineering, Moutai Institute, Renhuai 564501, China

**Keywords:** auxiliary metabolic genes, metagenomics, solid-state brewing, traditional Chinese vinegar, virus diversities

## Abstract

Traditional Chinese vinegar offers an exceptional flavor and rich nutrients due to its unique solid-state fermentation process, which is a multiple microbial fermentation system including various bacteria, fungi and viruses. However, few studies on the virus diversities in traditional Chinese vinegar have been reported. In this paper, using Zhenjiang aromatic vinegar as a model system, we systemically explored the viral communities in the solid-state brewing process of traditional Chinese vinegar using bacterial and viral metagenomes. Results showed that the viral diversity in vinegar *Pei* was extensive and the virus communities varied along with the fermentation process. In addition, there existed some interactions between viral and bacterial communities. Moreover, abundant antibiotic resistance genes were found in viromes, indicating that viruses might protect fermentation bacteria strains from the stress of antibiotics in the fermentation environment. Remarkably, we identified abundant auxiliary carbohydrate metabolic genes (including alcohol oxidases, the key enzymes for acetic acid synthesis) from viromes, implying that viruses might participate in the acetic acid synthesis progress of the host through auxiliary metabolic genes. Taken together, our results indicated the potential roles of viruses in the vinegar brewing process and provided a new perspective for studying the fermentation mechanisms of traditional Chinese vinegar.

## 1. Introduction

Traditional Chinese vinegar has played an essential role in Chinese life through the ages for its unique flavor, high nutrition (such as polyphenols, organic acids, melanoidins and tetramethylpyrazine) and many important biological functions, including antioxidative activity, liver protection, blood pressure and glucose control [1], lipid metabolism regulation and anti-tumor [2]. The main characteristic of traditional Chinese vinegar is the unique multispecies solid-state fermentation process, which is conducted in an open fermentation environment containing many microorganisms, including viruses, bacteria and fungi [3]. Presently, there were many studies reported on the bacteria and fungi in traditional Chinese vinegar fermentation, but few studies focused on viruses.

Viruses are hypothesized to be a major driver of their hosts’ evolution [4]. In general, viruses such as bacteriophages are considered harmful to fermented food production by decreasing the fermentative capacity of fermentative strains, occasionally resulting in complete fermentation failure. However, the diversity of viruses varied in diverse fermented foods due to the different environments. It has been observed that some viruses can positively influence the fermentation process of fermented foods [5]. Reports showed that, in some fermented food such as cocoa beans and milk cheese, viruses can regulate bacterial community succession during fermentation, and might have beneficial effects on the quality and sensory characteristics of fermented products [6,7]. Moreover, Pacini and Ruggiero [8] suggested that phages have potential probiotic properties in modern fermented foods and fermented milk supplemented with probiotics bacteriophage can improve the efficacy of probiotics in food. In traditional Chinese vinegar, viruses participate in the fermentation progress due to the open fermentation environment and might play important roles in fermentation. However, the effects of viruses on traditional Chinese vinegar fermentation remain unclear.

Viruses could encode a series of homologous genes related to host metabolism, which are named auxiliary metabolic genes (AMGs) [9]. Virus-encoded AMGs were regarded as one of the main ways by which viruses manipulated their hosts’ metabolism [10,11]. Reports showed that virus-encoded AMGs could reprogram specific host metabolic pathways, including maintaining, driving or short-circuiting key steps of host metabolic processes [10]. Most of the known virus-encoded AMGs are mainly involved in the host photosynthesis [12], carbon metabolism [13], nutrients’ cycling (such as nitrogen, phosphorus and sulfur) [10,14] and nucleotide biosynthesis process [12,15]. In addition, recent studies have reported that virus-encoded AMGs can also participate in the host’s response to abiotic stress. Viral metagenomics analysis indicated that the lysogenic phages encoded more AMGs under the stress of chromium to regulate hosts’ detoxification of heavy metal [16]. Studies on soil environmental viruses showed that, with the increase of pesticide stress, the diversity and abundance of virus-encoded AMGs associated with pesticides degradation elevated significantly, thus protecting the host from pesticide stress [17]. In contrast to the studies of virus communities in other ecosystems, the diversity and functional roles of virus-encoded AMGs in fermented foods, especially in traditional Chinese vinegar, are still unknown.

In the present study, using Zhenjiang aromatic vinegar (one of the four famous vinegar in China) as a model system, we systemically explore the viral communities in the solid-state brewing process of traditional Chinese vinegar. Firstly, the virus community structure in vinegar *Pei* and the rules concerning its changes with acetic acid fermentation progress were analyzed using viral metagenomes. Then, using bacterial and viral metagenomes, the interaction between virus and bacteria during vinegar fermentation was investigated. Furthermore, the AMGs encoded by viruses in vinegar *Pei* were analyzed, especially AMGs related to acetic acid metabolism. This study provides a new perspective for studying traditional Chinese vinegar fermentation mechanisms.

## 2. Materials and Methods

### 2.1. Vinegar Pei Samples Collection

**Figure 1:**
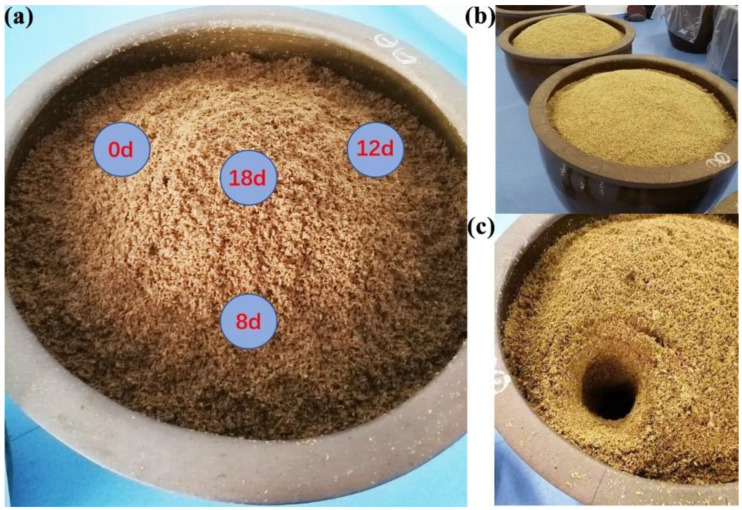
Vinegar *Pei* sample points distributed in the ceramic vat. (**a**) The 0th, 8th, 12th sample points were distributed at the tri-sector (about 15 cm from the wall) of the ceramic vat. The 18th was distributed at the center of the ceramic vat. (**b**) There were three biological replicates for each sample point. (**c**) In every sampling station, vinegar *Pei* from top to bottom in ceramic vat were took out and well mixed.

The acetic acid fermentation process of traditional Chinese vinegar was carried out in uncovered ceramic vats (height: 1.11 m, diameter: 0.8 m) in an open fermentation environment from April to June 2021. Vinegar *Pei* samples for metagenomic analysis, the primary raw material of traditional Chinese vinegar brewing in the acetic acid fermentation process, were obtained before turning over vinegar *Pei* on the 0th, 8th, 12th and 18th day of fermentation, respectively. The sample points were distributed at the tri-sector (about 15 cm from the wall) of the ceramic vat, as shown in Figure 1, avoiding the influence of repeated sampling on virus communities at the same sampling site. To fully reflect the virus communities in the whole ceramic vats, according to the method of Kou [18], vinegar *Pei* samples from top to bottom in the ceramic vat were taken out and well mixed at every sample point (Figure 1c), and then reduced by coning and quartering repeatedly; about 500 g vinegar *Pei* samples were obtained and stored at −80 °C until use. There were three biological replicates for each sampling site (Figure 1b).

### 2.2. Virus Purification

The viruses in vinegar *Pei* were purified according to the method of Dugat-Bony [19] with slight modification. Briefly, 5 g vinegar *Pei* samples were put into a sterile bag. Then, 50 mL cold sterile 2% (*w*/*v*) trisodium citrate was added and mixed for 3 min using a flapping sterile homogenizer (Shanghai Lichen Bangxi Instrument Technology Co., Ltd., Shanghai, China). After centrifuging at 500× *g*, 4 °C for 10 min, big aggregates in the solution were discarded. The supernatant was subsequently centrifuged at 5000× *g*, 4 °C for 10 min to remove the microbial cells and the free viral particles were in the supernatant. Then, the precooled SM buffer (200 mM NaCl, 10 mM MgSO_4_, 50 mM Tris pH 7.5) supernatant was used to dilute the supernatant in a ratio of SM buffer: supernatant = 5:1 (*v*/*v*). Next, the solution was filtrated using 0.22 μm polyethersulfone membranes. RNase A and DNase I were added into the filtrate with the final concentration of 1 μg/mL and left at 37 °C for 30 min to degrade genomic DNA and RNA of bacterial cells. Then, cold sterile PEG 8000 (Sigma) aqueous solution was added to the solution to make a final concentration 10% (*w*/*v*) and kept overnight at 4 °C to precipitate viral particles. The solution was subsequently centrifuged at 12,000× *g*, 4 °C for 1 h and the supernatant was discarded. Finally, virus particles in bottom sediment were resuspended with 2 mL of cold SM buffer and then stocked at 4 °C until ready to use.

### 2.3. Viral and Bacterial DNA Extraction and Virome Sequencing

Genomic DNA of purified viruses in vinegar *Pei* was extracted using a Magnetic Virus DNA/RNA Kit (Tiangen Biotech (Beijing) Co., Ltd., Beijing, China) following the manufacturer’s protocol. Genomic DNA of bacteria in vinegar *Pei* was extracted using a TIANamp Bacteria DNA Kit (Tiangen Biotech (Beijing) Co., Ltd., Beijing, China) following the manufacturer’s protocol. DNA libraries were constructed with an insert size of 300 bp and sequenced using an Illumina Hiseq PE150 sequencing platform (Illumina Inc., San Diego, CA, USA) at the Shanghai Rongxiang Biotechnology Co., Ltd. (Shanghai, China). Raw data produced were filtered to remove reads containing “N” bases (N parameter setting 10), reads with adaptors and low-quality reads with a quality score of <20 using Cutadapt software version 1.18 [20], retaining high-quality clean data for subsequent analysis. All virome reads were assembled into contigs using Megahit software version 1.1.3 [21]. Moreover, open reading frames (ORFs) were predicted using MetaGeneMark [22].

### 2.4. Identification of Auxiliary Carbohydrate Metabolic Genes

According to the method of Jin [23], in order to obtain the clusters of orthologous groups of proteins (COG) corresponding to vinegar *Pei* virus genes, COG functional annotation of vinegar *Pei* viromes was performed using protein–protein BLAST (BLASTp) software [24]. Predicted ORFs of vinegar *Pei* viromes were compared with eggNOG database (http://eggnog5.embl.de/ (accessed on 30 November 2021)) with an e-value threshold of 1 × 10^−5^. Furthermore, ORFs related to carbohydrate metabolism of vinegar Pei viromes were obtained from ORFs belonging to the COG function class of carbohydrate transport and metabolism using CD-Hit software [25] with thresholds of 95% identity plus 90% coverage. Subsequently, carbohydrate-active enzymes (CAZymes) from these viral ORFs were identified by the *hmmscan* program from HMMER v.3.1 [26] compared with CAZymes database using e-value ≤ 1 × 10^−5^ as a cut-off. In addition, in order to receive the best annotation of each ORF, ORFs associated with carbohydrate metabolism and CAZymes were compared with the National Center for Biotechnology Information (NCBI) NR and Pfam database.

### 2.5. Antibiotic Resistance Gene Search

The CDS of bacterial and viral metagenomes ORFs were used as queries to search for ARGs in vinegar *Pei* virome using Diamond software [27] against the comprehensive antibiotic resistance database (CARD) with the comparison parameter was set to ‘strict’.

## 3. Results and Discussion

### 3.1. Investigation of Bacterial and Viral Metagenomes of Vinegar Pei

We defined the reads with a quality score of ≥20, no adaptors and no ambiguous ‘N’ bases as high-quality reads. In bacterial metagenomics, an average of 11.16 gigabase pairs (Gbp) with GC content of 55.58% and 76.25 million high-quality reads were obtained based on Q20% > 90. A total of 1,136,418 contigs were assembled from 12 DNA samples extracted from vinegar *Pei* bacteria (Table 1) and gene sequence length averaged 1044 bp (N50: 1193 bp). In viral metagenomics, an average of 1.6 Gbp with GC content of 54.43% and 11.05 million high-quality reads were acquired based on Q20% > 90. Gene sequence length averaged 1080 bp (N50: 1264 bp). Moreover, a total of 150,703 contigs were assembled from 12 DNA samples extracted from vinegar *Pei* virus (Table 1).

Moreover, we made an ORFs prediction analysis using MetaGeneMark software. One hundred and thirty-three thousand nine hundred and fifty-nine and twenty thousand five hundred and fifteen ORFs were obtained in metagenomics and viral metagenomics of vinegar *Pei* samples, respectively (Table 1). The above results showed that the throughput and sequencing quality of bacterial and viral metagenomes sequencing data of vinegar *Pei* were high enough for the following analyses.

### 3.2. Taxonomic Diversities of Vinegar Pei Viral and Bacterial Communities

**Figure 2:**
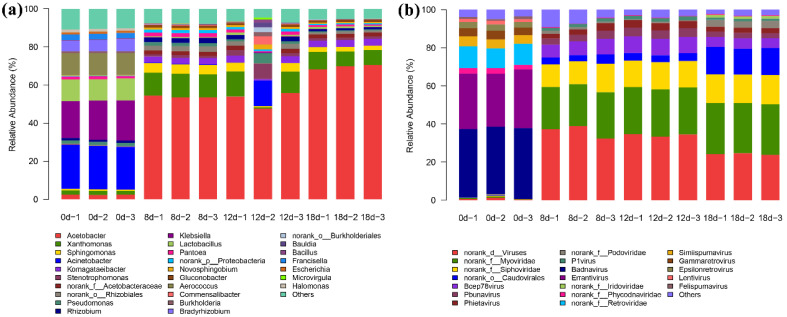
Bacterial and viral community diversities (at genus level) in vinegar *Pei* ((**a**) bacteria, (**b**) virus).

Bacterial taxonomic affiliations of vinegar *Pei* were determined by comparing the predicted bacterial genomic ORFs with bacterial sequences from the NCBI RefSeq Bacteria database. Results showed that a total of 532 bacterial families were identified in vinegar *Pei* during acetic acid fermentation (Supplementary File S1). *Acetomonas* accounted for the largest fraction, and the following in order were *Xanthomonaceae*, *Sphingomonas*, *Komagataeibacter* and *Oligotrophiaceae*. In addition, 1668 bacterial genera were identified in vinegar *Pei* (Figure 2a).

Furthermore, by comparing the virome ORFs to those in the NCBI RefSeq Virus database, the viral taxonomic composition of vinegar *Pei* was obtained. Results showed that only a tiny fraction of the virome ORFs was similar to some sequences in the NCBI RefSeq Virus database, while most of the vinegar *Pei* viruses were unknown. Finally, a total of 40 viral families were identified in vinegar *Pei* viruses (Supplementary File S2). *Myoviridae* accounted for the largest fraction in vinegar *Pei* and *Siphoviridae*, *Caudovirales* were, respectively, in the second and third position. In addition, 258 viral genera were identified in vinegar *Pei* (Figure 2b).

### 3.3. Dynamic Changes of Viral Communities during the Fermentation and the Interaction with Bacterial Community in Vinegar Pei

**Figure 3:**
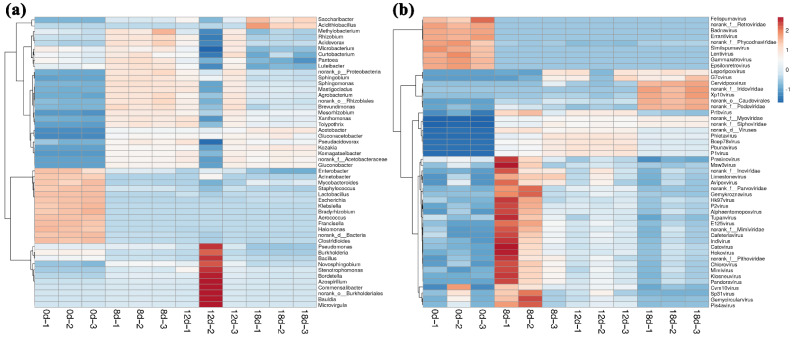
Dynamic changes in bacterial and viral community structure (at genus level) in vinegar *Pei* during acetic acid fermentation ((**a**) bacteria, (**b**) virus).

The virus communities in vinegar *Pei* varied with the fermentation process (Figure 3b). Compared with the fermentation at 0d, the virus community structure changed significantly after the beginning of fermentation. On day 0 of fermentation, *Badnavirus* and *Errantivirus* were the main two virus genus, which might be from the fermentation raw materials (rice wine, rice husk and bran), while, on the 8th day of fermentation, the top five virus genus were *Myoviridae*, *Siphoviridae*, *Caudovirales*, *Bcep78* and *Pbunavirus*. In addition, the community structure of viruses in vinegar *Pei* also changed along with fermentation. Compared with the 8th day of fermentation, the abundance of *Myoviridae* and *Siphoviridae* increased gradually, while *Badnavirus* and *Errantivirus* decreased and were not detected on the 18th day of fermentation. *Bcep78*, *Pbunavirus* and *Phietavirus* increased firstly, then reduced. *Podoviridae* decreased firstly and then increased. *Caudovirales* remained unchanged at first and gradually increased on the 12th day of fermentation. However, the change in *P1 virus* was insignificant.

To better understand the interactions between virus and bacteria in vinegar *Pei*, the changes of bacterial community structure during acetic acid fermentation were also analyzed using metagenomics. It was found that from the 8th day of fermentation, *Acetomonas* was the dominant bacteria. According to the abundance value, the top five genera were *Acetomonas*, *Xanthomonas*, *Sphingomonas*, *Komagataeibacter* and *Stenotrophomonas*. Among them, *Acetomonas* was the main dominant bacteria. By the 18th day of fermentation, the abundance of *Acetomonas* reached the maximum (78.4%). The changes in virus community and microbial community were preliminarily analyzed. The results showed that *Myoviridae*, *Siphoviridae* and *Caudovirales* were consistent with the growth trend of *Acetomonas*, while *Badnavirus* and *Errantivirus* showed the opposite trend to that of *Acetomonas*, indicating that *Myoviridae*, *Siphoviridae*, *Caudovirales*, *Badnavirus* and *Errantivirus* might play an essential role in the changes of *Acetomonas* community and metabolism. Consistently, Koki Omata [28] obtained some temperate phages from acetic acid bacteria induced by mitomycin C, and transmission electron microscopy and genomic analysis revealed that all of these belong to the myoviridae-type phage, indicating that acetic acid bacteria was the host of the myoviridae-type phage and the latter might affect the metabolism of acetic acid bacteria.

### 3.4. Abundant Auxiliary Carbohydrate Metabolic Genes in Vinegar Pei Viruses

**Figure 4:**
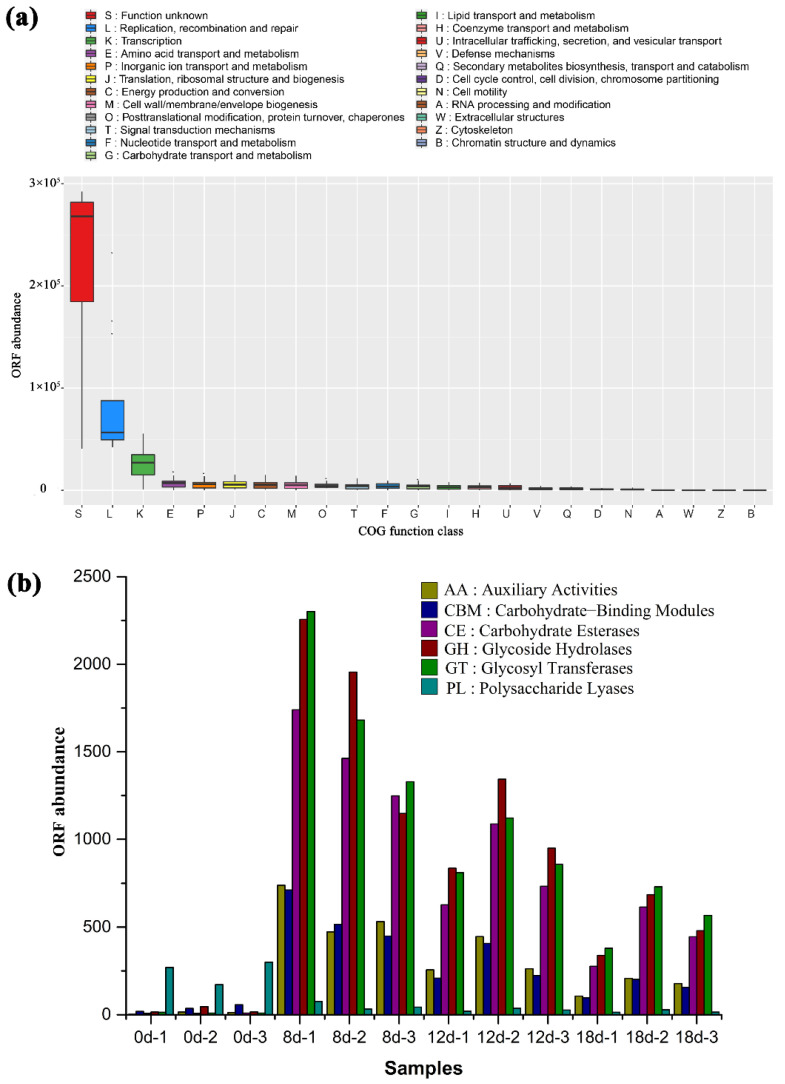
Abundant auxiliary carbohydrate-metabolism genes in vinegar *Pei* viruses. (**a**) COG functional annotation of vinegar *Pei* virome. (**b**) Annotation of viral carbohydrate-metabolism related ORFs in the CAZymes database.

Viruses could regulate host metabolism by encoding auxiliary metabolic genes (AMGs), a series of homologous genes related to host metabolism [10,12]. Moreover, AMGs encoded by viruses have different characteristics and advantages from host homologous genes [29]. The raw materials of traditional Chinese vinegar are mainly carbohydrate-rich cereals such as rice and sorghum [2], and there are many carbohydrate catabolism and anabolism reactions that happen in traditional Chinese vinegar brewing, which need many carbohydrate-metabolism enzymes. In order to investigate whether vinegar *Pei* viruses contain auxiliary carbohydrate metabolic genes or not, vinegar *Pei* virome sequences were compared with the eggNOG database to obtain the clusters of orthologous groups of proteins (COG) corresponding to the virus genes using BLASTp. COG functional classification in Figure 4a showed that most viral ORFs were not annotated, revealing that there were a large number of uncharacterized viral genes in vinegar *Pei*. Although annotated viral ORFs were grouped into all COG functional categories, most ORFs were associated to conventional viral functions such as ‘replication, recombination and repair’, ‘amino acid transport and metabolism’, ‘translation, ribosomal structure and biogenesis’, ‘energy production and conversion’, ‘nucleotide transport and metabolism’. Notably, besides the above-mentioned, COG functional categories for ‘carbohydrate transport and metabolism’ were significantly over-represented in vinegar *Pei* virome (Figure 4a).

In the carbohydratae-active enzyme (CAZymes) database, carbohydrate-active enzymes from different species can be separated as glycoside hydrolases (GHs) family, glycosyltransferases (GTs) family, polysaccharide lyases (PLs) family, carbohydrate esterases (CEs) family, carbohydrate-binding modules (CBMs) family and auxiliary activities (AAs) family. Using *hmmscan* program from HMMER v.3.1 software, the vinegar *Pei* viral ORFs were compared with the CAZymes database. Results showed that, in vinegar *Pei* viruses, CEs, GHs and GTs family were the top three auxiliary carbohydrate-metabolism genes, and the highest gene abundance was obtained on the 8th day of fermentation (Figure 4b). Consistently, the total acid contents of vinegar *Pei* increased rapidly from the 8th day of fermentation (Supplementary Figure S1), indicating that fermentation metabolism became vigorous from the 8th day of fermentation, requiring more carbohydrate-metabolism enzymes to produce more acetic acid.

Moreover, a total of 127 ORFs were further identified as CAZymes, while 43 auxiliary carbohydrate-metabolism genes were identified in vinegar *Pei* viruses, which belong to AAs, CEs, GHs, GTs and PLs family, including the common alcohol oxidases, acetylesterase, cellulase, peptidoglycan lytic transglycosylases, mannanase, chitinase, glucosyltransferase, starch phosphorylase, glucan synthase and pectate lyase (Table 2). Among them, alcohol oxidases are key enzymes in acetic acid synthesis [30]. Cellulose-rich materials such as wheat bran and rice hulls are raw materials of Zhenjiang aromatic vinegar in the acetic acid fermentation process. Meanwhile, Takahashi reported that cellulase might increase the output of vinegar [31]. Presently, the results implied that viruses in vinegar *Pei* might play essential roles in acetic acid metabolisms through these auxiliary carbohydrate metabolic genes in the acetic acid fermentation process of traditional Chinese vinegar. Consistently, auxiliary carbohydrate metabolic genes were also found in viruses from other ecosystems [23]. The latest research showed that virus-encoded genes related to carbon metabolism (*talC*, *cp12*) were found in a cyanophage S-SZBM1 [13].

### 3.5. Abundant Antibiotic Resistance Genes in Vinegar Pei Viruses

Viruses were considered as crucial reservoirs of antibiotic resistance genes in the environment [32]. A recent study on the river ecosystem reported that bacteriophages played an important role in antibiotic resistance genes dissemination [33]. Presently, whether ARGs existed in vinegar *Pei* viromes was also investigated. Results showed that ARGs were obtained in vinegar *Pei* viromes, which mainly included genes for aminoglycoside, novobiocin, multidrug transport proteins, polymyxin, sulfonamide, fluoroquinolone, elfamycin, mupirocin and quinolone resistance proteins (Table 3).

Aminoglycoside resistance proteins function mainly by inactivating aminoglycosides via enzymatic modification of the antibiotic chemical structure [34] or preventing aminoglycosides from binding to the ribosome [35]. Novobiocin resistance proteins found in vinegar *Pei* viruses were alanyl-tRNA synthetase (alaS). Multidrug transport proteins were antibiotic efflux complex, including ABC-type multidrug efflux pump components [36,37,38]. Polymyxin resistance proteins including ArnA, ArnC and ArnT are required for the synthesis and transfer of 4-amino-4-deoxy-L-arabinose (Ara4N) to Lipid A. Previous reports showed that one of the mechanisms of polymyxins’ resistance was the regulatory network controlling chemical modifications of lipid A moiety anchored on lipopolysaccharide, reducing the negative charge of lipid A and its affinity for polymyxins [39]. Sulfonamide resistance proteins were sulfonamide resistant dihydropteroate synthase, which was encoded by two known genes sulI and sulII [40]. Meanwhile, genes at low frequencies encoding resistance proteins for fluoroquinolone, elfamycin, mupirocin and quinolone were also obtained in the vinegar *Pei* viromes (Table 3).

Overall, the proportions of ARGs in vinegar *Pei* viral communities ranged from approximately 0 to 0.0062% (Table 3). The proportions of ARGs in the vinegar *Pei* viromes were comparable with ARG levels of viruses detected in other studies. Bioinformatics analysis of public data downloaded from NCBI RefSeq Protein Database showed that the mean proportion of predicted ARGs found in prophages from natural environments was 0–0.0028% [32]. Moreover, a more variable but still low proportion (0.07–0.12%) of virome reads were annotated as ARGs in the river ecosystem viromes [36].

By comparing the predicted ORFs of the viral contigs against the Comprehensive Antibiotic Resistance Database (CARD), ARGs in vinegar *Pei* viromes were obtained. Results showed that a total of 29 ARGs were found in vinegar *Pei* viromes (Table 4). Some ARGs conferred antibiotic resistance by forming an antibiotic efflux complex. lrfA, which is a well-characterized *M. smegmatis* efflux pump [41] and is involved in the active efflux of quinolones, was found in five vinegar *Pei* viral contigs. Multidrug transport proteins macA were found in six vinegar *Pei* viral contigs and macB were found in two vinegar *Pei* viral contigs. Moreover, *APH (3’)-IIc*, encoded by the ORFs 12d-2-k141-17472 gene 21,035 and 12d-2-k141-18203 gene 22034, is a chromosomal-encoded aminoglycoside phosphotransferase [42] and could inactivate aminoglycoside. Some ARGs, such as EF-Tu, parC and gyrB, conferred antibiotic resistance through antibiotic resistant gene variants or mutants (Table 4). Additionally, alanyl-tRNA synthetase (alaS), an aminocoumarin resistance gene, were found in eight vinegar *Pei* viral contigs. IleS, a mupirocin resistance gene, was found in three vinegar *Pei* viral contigs.

Moreover, datasets for bacterial community and metagenomes in vinegar *Pei* were also obtained in parallel at identical sampling stations. Results showed that the ARGs genes above-mentioned were also detected in bacterial metagenomes (Supplementary File S3), revealing that fermentation bacteria strains might be protected by virus-encoded ARGs genes from the stress of antibiotics in the fermentation environment.

## 4. Conclusions

Studies on the microenvironment of the solid-state fermentation process are very important for clarifying the fermentation mechanisms of traditional Chinese vinegar. In the present study, using Zhenjiang aromatic vinegar as a model system, we systemically explored the viral communities in the solid-state brewing process of traditional Chinese vinegar for the first time. The results revealed extensive viral diversity in vinegar *Pei*, which varied with the fermentation process. Meanwhile, there existed some interactions between viral and bacterial communities in vinegar *Pei*. Moreover, we identified abundant antibiotic resistance genes and auxiliary carbohydrate metabolic genes (including alcohol oxidases, the key enzymes in acetic acid synthesis) from vinegar *Pei* viromes, indicating the potentially important roles of viruses in traditional Chinese vinegar brewing. In a word, our results provided a new perspective for studying the fermentation mechanisms of traditional Chinese vinegar. However, there is still so much work to be undertaken. Primarily, viruses encoding AMGs in traditional Chinese vinegar brewing would need to be isolated and the functions of these AMGs in the acetic acid fermentation process would need to be verified in the future.

## Figures and Tables

**Table 1:** Investigation of bacterial and viral metagenomics of vinegar *Pei*.

Samples	Bacterial Metagenomes				Viral Metagenomes			
	Number of Clean Reads	Q20%	Number of Contigs	Predicted ORF Number	Number of Clean Reads	Q20%	Number of Contigs	Predicted ORF Number
0d-1	61,503,358	98.23	84,185	57,456	15,100,950	96.54	5169	3224
0d-2	65,015,640	98.19	87,846	60,122	7,127,744	96.64	1002	626
0d-3	62,704,114	98.09	124,169	85,929	31,426,924	97.05	24,646	16,876
8d-1	106,916,406	97.53	141,863	230,229	4,713,670	93.42	15,783	27,164
8d-2	84,764,580	91.23	128,904	208,635	4,543,128	91.23	16,241	27,219
8d-3	103,547,844	97.26	142,724	233,312	12,537,328	93.49	15,203	36,663
12d-1	84,039,874	96.89	102,676	175,735	9,204,336	93.89	15,562	26,444
12d-2	17,228,660	96.63	18,150	43,128	17,228,660	93.73	18,183	43,145
12d-3	96,679,336	96.29	126,429	212,888	6,556,868	93.41	14,005	22,960
18d-1	61,944,434	97.27	53,651	90,339	12,858,800	93.46	10,056	17,343
18d-2	94,410,756	97.48	72,331	121,053	5,626,462	93.46	7917	13,722
18d-3	76,201,500	97.42	53,490	88,684	5,692,556	91.30	6936	10,804

The meaning for d-1, d-2, d-3 is three biological replicates.

**Table 2:** Annotated auxiliary CAZymes from vinegar *Pei* viruses.

Viral Auxiliary CAZymes	Viral Auxiliary CAZymes
AAs family	Peptidoglycan hydrolase with Endo-beta-N-acetylglucosaminidase specificity (GH73)
Copper-dependent lytic polysaccharide monooxygenases (AA10)	Amylomaltase or 4-alpha-glucanotransferase (GH77)
Pyrroloquinoline quinone-dependent oxidoreductase (AA12)	Alpha-L-rhamnosidase (GH78)
Class II lignin-modifying peroxidases (AA2)	Chitosanase (GH80)
Glucose-methanol-choline (GMC) oxidoreductases (AA3)	Glycoprotein endo-alpha-1,2-mannosidase (GH99)
Vanillyl-alcohol oxidases (AA4)	GTs family
1,4-benzoquinone reductases (AA6)	Lipid-A-disaccharide synthase (GT19)
CEs family	Cellulose synthase (GT2)
UDP-3-0-acyl N-acetylglucosamine deacetylase (CE11)	Alpha, alpha-trehalose-phosphate synthase (GT20)
Acetylesterase (CE16)	Ceramide beta-glucosyltransferase (GT21)
Acetyl xylan esterase (CE2, CE3, CE6)	Polypeptide alpha-N-acetylgalactosaminyltransferase (GT27)
GHs familyCellulase (GH5)	Alpha-3-deoxy-D-manno-octulosonic-acid (KDO) Transferase (GT30)
Peptidoglycan lytic transglycosylases (GH23, GH102, GH103, GH104)	Glycogen or starch phosphorylase (GT35)
Unsaturated rhamnogalacturonyl hydrolase (GH105)	Peptide beta-N-acetylglucosaminyltransferase (GT41)
N-acetylmuramidase (GH108)	Murein polymerase (GT51)
Alpha-N-acetylgalactosaminidase (GH109)	Lipid II Fuc4NAc transferase (GT56)
Beta-mannanase (GH113)	Alpha-3-deoxy-D-manno-octulosonic-acid (KDO) Transferase (GT73)
Endo-alpha-1,4-polygalactosaminidase (GH114)	Cyclic beta-1,2-glucan synthase (GT84)
β-L-arabinofuranosidase (GH127)	PLs family
Chitinase (GH19)	Pectate lyase (PL10)
Lysozyme (GH24, GH25)	Heparin-sulfate lyase (PL12)
Alpha-L-fucosidase (GH29)	Oligo-alginate lyase (PL15)
Alpha, alpha-trehalase (GH37)	Alginate lyase (PL17, PL5)
Beta-agarase (GH50)	Oligogalacturonate lyase/oligogalacturonide lyase (PL22)

**Table 3:** Number of virome reads that were predicted to confer antibiotic resistance.

Target Antibiotics	0d-1	0d-2	0d-3	8d-1	8d-2	8d-3	12d-1	12d-2	12d-3	18d-1	18d-2	18d-3
sulfonamide	0	0	0	36	23	5	8	3	10	2	11	3
multidrug transport	0	0	0	49	54	41	18	36	23	9	21	19
aminoglycoside	0	0	1	53	39	40	12	37	20	5	11	13
fluoroquinolone	0	0	0	9	11	10	3	9	3	1	4	4
elfamycin	0	0	0	14	11	20	7	14	5	3	6	4
polymyxin	0	0	0	29	30	26	10	20	14	5	10	11
mupirocin	0	0	0	13	12	9	4	8	4	1	2	3
novobiocin	0	0	0	69	37	21	13	14	15	8	12	20
quinolone	0	0	0	20	8	19	5	17	5	2	2	3
Total	0	0	1	292	225	191	80	158	99	36	79	80
% in virome reads ^a^	0	0	0	0.0062%	0.005%	0.0015%	0.0009%	0.0009%	0.0015%	0.0003%	0.0014%	0.0014%

^a^ Total number of reads predicted to confer antibiotic resistance/total number of virome reads assigned a known function ×100. The meaning for d-1, d-2, d-3 is three biological replicates.

**Table 4:** List of antibiotic resistance genes retrieved from vinegar *Pei* virome contigs.

Protein ID ^a^	ARO Category ^b^	ARG Name	*e* Value	% Identity
12d-2-k141-25303 gene 30071	efflux pump	lrfA	2.19 × 10^−33^	27.59
12d-2-k141-25303 gene 30077	efflux pump	lrfA	7.04 × 10^−33^	30.08
12d-2-k141-8532 gene 10306	efflux pump	lrfA	2.29 × 10^−67^	37.23
12d-2-k141-23839 gene 28370	efflux pump	lrfA	2.41 × 10^−39^	31.50
8d-3-k141-5725 gene 7838	efflux pump	lrfA	1.02 × 10^−66^	37.23
18d-1-k141-10283 gene 7834	efflux pump	macA	1.62 × 10^−52^	47.12
12d-2-k141-15026 gene 18130	efflux pump	macA	2.48 × 10^−64^	37.40
12d-2-k141-13619 gene 16396	efflux pump	macA	1.72 × 10^−64^	36.41
12d-1-k141-16328 gene 12997	efflux pump	macA	3.76 × 10^−85^	39.53
12d-2-k141-26375 gene 31413	efflux pump	macA	7.51 × 10^−84^	41.19
12d-2-k141-28030 gene 33312	efflux pump	macA	1.28 × 10^−81^	41.58
18d-3-k141-6633 gene 4643	efflux pump	macB	8.90 × 10^−133^	54.19
8d-3-k141-28586 gene 37717	efflux pump	macB	6.36 × 10^−161^	40.54
12d-2-k141-17472 gene 21035	antibiotic inactivation	APH (3’)-IIc	2.77 × 10^−161^	84.64
12d-2-k141-18203 gene 22034	antibiotic inactivation	APH (3’)-IIc	8.53 × 10^−153^	80.52
12d-1-k141-16136 gene 12816	antibiotic resistant gene variant or mutant	EF-Tu	1.27 × 10^−109^	70.56
8d-3-k141-1508 gene 1984	antibiotic resistant gene variant or mutant	parC	6.32 × 10^−119^	49.74
8d-3-k141-4044 gene 5547	antibiotic resistant gene variant or mutant	gyrB	5.97 × 10^−171^	47.53
12d-3-k141-25307 gene 18959	aminocoumarin resistance gene	alaS	5.88 × 10^−167^	66.57
8d-2-k141-36863 gene 29116	aminocoumarin resistance gene	alaS	5.04 × 10^−118^	87.57
18d-1-k141-10925 gene 8326	aminocoumarin resistance gene	alaS	1.61 × 10^−63^	55.17
8d-1-k141-631 gene 476	aminocoumarin resistance gene	alaS	8.61 × 10^−78^	62.96
8d-1-k141-34699 gene 28239	aminocoumarin resistance gene	alaS	8.77 × 10^−110^	83.62
8d-3-k141-6438 gene 8856	aminocoumarin resistance gene	alaS	8.53 × 10^−97^	61.64
8d-2-k141-3704 gene 3047	aminocoumarin resistance gene	alaS	1.78 × 10^−167^	59.60
18d-3-k141-15560 gene 10446	aminocoumarin resistance gene	alaS	5.77 × 10^−57^	59.12
8d-1-k141-5267 gene 4197	mupirocin resistance gene	ileS	1.13 × 10^−59^	24.94
12d-1-k141-32581 gene 25448	mupirocin resistance gene	ileS	9.06 × 10^−58^	25.50
8d-3-k141-12723 gene 17311	mupirocin resistance gene	ileS	2.07 × 10^−64^	25.68

^a^ Query sequence name of viral ORFs in vinegar *Pei* samples. ^b^ ARG classification in CARD database.

## Data Availability

The raw data of bacterial and viral metagenomes are available at NCBI Sequence Read Archive (NCBI SRA) under BioProject accession no. PRJNA884748. http://www.ncbi.nlm.nih.gov/bioproject/884748. The accession numbers of vinegar *Pei* samples deposited in the NCBI SRA database are listed in Supplementary File S4.

## Supplementary Materials

The following supporting information can be downloaded at: https://www.mdpi.com/article/10.3390/foods11203296/s1. Supplementary Figure S1: Changes of total acid contents in vinegar *Pei* during the acetic acid fermentation process of traditional Chinese vinegar; Supplementary File S1: Bacterial families identified in vinegar *Pei* during acetic acid fermentation; Supplementary File S2: Viral families identified in vinegar *Pei* during acetic acid fermentation; Supplementary File S3: ARGs genes detected in bacterial metagenomes; Supplementary File S4: The accession numbers of vinegar *Pei* samples deposited in the NCBI SRA database.
