# Supplementary material for: Metagenomics of Virus Diversities in Solid-State Brewing Process of Traditional Chinese Vinegar
Source: Foods. 2022 Oct 21;11(20):3296. doi: 10.3390/foods11203296 (PMC9602057; doi:10.3390/foods11203296)
Supplement: Supplementary file 1 [file foods-11-03296-s001.zip › Supplementary Figure S1.pdf]

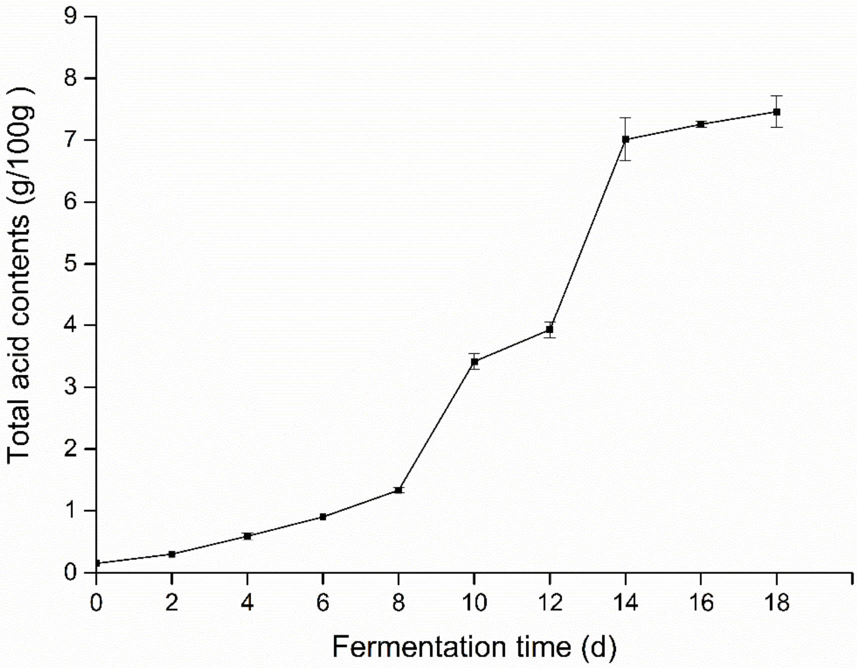

Figure S1. Changes of total acid contents in vinegar *Pei* during the acetic acid fermentation process of traditional Chinese vinegar.
