# Supplementary material for: Metagenomics of Virus Diversities in Solid-State Brewing Process of Traditional Chinese Vinegar
Source: Foods. 2022 Oct 21;11(20):3296. doi: 10.3390/foods11203296 (PMC9602057; doi:10.3390/foods11203296)
Supplement: Supplementary file 1 [file foods-11-03296-s001.zip › Supplementary file S4.pdf]

The raw sequencing data of bacterial and viral metagenomes of vinegar *Pei* samples in this study were deposited in the NCBI SRA database under the accession numbers listed in the table below.

Sample name	SRA ID	Sample name	SRA ID
0d-bacterial-1	SRR21774940	0d-viral-1	SRR21774936
0d-bacterial-2	SRR21774939	0d-viral-2	SRR21774935
0d-bacterial-3	SRR21774928	0d-viral-3	SRR21774934
8d-bacterial-1	SRR21774923	8d-viral-1	SRR21774933
8d-bacterial-2	SRR21774922	8d-viral-2	SRR21774932
8d-bacterial-3	SRR21774921	8d-viral-3	SRR21774931
12d-bacterial-1	SRR21774920	12d-viral-1	SRR21774930
12d-bacterial-2	SRR21774919	12d-viral-2	SRR21774929
12d-bacterial-3	SRR21774918	12d-viral-3	SRR21774927
18d-bacterial-1	SRR21774917	18d-viral-1	SRR21774926
18d-bacterial-2	SRR21774938	18d-viral-2	SRR21774925
18d-bacterial-3	SRR21774937	18d-viral-3	SRR21774924
